# Effect of Temperature, Concentration and Contact Time of Sodium Hypochlorite on the Treatment and Revitalization of Oral Biofilms

**DOI:** 10.15171/joddd.2015.038

**Published:** 2015-12-30

**Authors:** Aldo del Carpio-Perochena, Clovis Monteiro Bramante, Marco Hungaro Duarte, Flaviana Bombarda de Andrade, Marcia Zardin Graeff, Marina Marciano da Silva, Bruno Cavalini Cavenago, Samuel Lucas Fernandes

**Affiliations:** ^1^Doctor of Sciences, Department of Endodontics, Bauru Dental School of Bauru, University of São Paulo, Bauru, São Paulo, Brazil; ^2^Associate Professor, Department of Endodontics, Bauru Dental School of Bauru, University of São Paulo, Bauru, São Paulo, Brazil; ^3^Laboratory Specialist, Integrated Research Centre (CIP), Bauru Dental School of Bauru, University of São Paulo, Bauru, São Paulo, Brazil; ^4^Master of Sciences, Department of Endodontics, Bauru Dental School of Bauru, University of São Paulo, Bauru, São Paulo, Brazil

**Keywords:** Bacteria, biofilms, dentin, sodium hypochlorite, temperature

## Abstract

***Background and aims.*** Increasing the temperature of sodium hypochlorite (NaOCl) enhances its dissolution and antibacterial properties. However, the high resistance of multi-species biofilms could restrict the effect of the solution regardless of its temperature, enabling the long-term recovery of the surviving bacteria. The aim of this study was to investigate if the increase of temperature of NaOCl improves its antibacterial and dissolution ability on oral biofilms and if the post-treatment remaining bacteria were capable of growing in a nutrient-rich medium.

***Materials and methods.*** Forty dentin blocks were infected intra-orally for 48 hours. Then, the specimens were treated with 1% and 2.5% NaOCl at room temperature (22ºC) and body temperature (37ºC) for 5 and 20 min. The percentage of live cells and the biovolume were measured pre- (control) and post-treatment and after the biofilm revitalization. Four confocal ‘stacks’ were chosen from random areas of each sample. Statistical analysis was performed using Kruskal-Wallis and Dunn tests. Statistical significance was defined at P <0.05.

***Results.*** All the NaOCl groups were effective in dissolving the biofilm at any temperature, concentration and contact time without statistical differences among them (P >0.05). The 1%-NaOCl for 5min was not able to significantly kill the bacteria, regardless of its temperature and contact time (P >0.05).

***Conclusion.*** The temperature variation of the NaOCl was not relevant in killing or dissolving bacterial biofilms. Twenty-four hours of reactivation did not appear to be enough time to induce a significant bacterial growth.

## Introduction

 In order to eliminate biofilms as far as possible from the root canal system during endodontic treatment, mechanical instrumentation and the applications of chemical solutions are used,^1^ such as NaOCl, chlorhexidine, ethylenediaminetetraaceticacid (EDTA) and others. Among all existing endodontic irrigants, NaOCl is the solution most recommended in endodontic therapy, due to its great tissue dissolution capacity and antibacterial effect.^2^

 In addition, although NaOCl is biologically acceptable when it is confined within the canal, it is highly caustic when it is extravasated beyond the apical region.^3^ This condition is aggravated as the solution concentration is increased. So, considering the caustic potential of NaOCl, it would not be advisable to use the solution in a high concentration. As an attempt to resolve this limitation, increasing the temperature of the NaOCl in low concentrations was proposed because it seems to enhance the dissolution and antibacterial properties of the irrigant. Furthermore, the toxicity of preheated NaOCl in a low concentration should be lower than the same solution in a high concentration at room temperature because the properties of both should be similar in the root canal when the solution achieves the body temperature.^4^ Although, there is almost a general consensus that increasing the NaOCl temperature improves its antibacterial and dissolution properties, there is little information in the endodontic literature on this subject.^4,5^

 At the time of conducting this research, it was possible to find nine studies on the antibacterial and dissolution effects of pre-heated NaOCl in PubMed search.^4-9^ Nonetheless, eight of them focusedon evaluating either dissolution,^5-10^ or antibacterial effects,^11,12^ and only one analyzed both effects.^4^ In addition, all the publications mentioned above were carried out in laboratory conditions using human or bovine pulps, bovine muscle, rat dermal connective tissue and collagen matrices to study the NaOCl dissolution ability; and uni-species biofilms to study the antibacterial capacity of NaOCl. Therefore, the authors consider that analyzing the effect of NaOCl in different temperatures on poly-microbial biofilms formed *in situ* offers a significant approximation with the *in vivo* biofilms, such as those formed in a necrotic root canal.

 On the other hand, the supply of nutrients to the bacteria in the oral cavity is one of the most abundant in comparison with other ecosystems because the host consumes food. However, in microenvironments such as root canals, the bacteria develop efficient metabolic adaptive mechanisms in order to survive under conditions of stress, nutrient scarcity or nutrient deprivation.^13^ These adaptive mechanisms alter the bacterial metabolism from biosynthesis and reproduction toward obtaining energy for its existing biological functions at that moment.^14^ Thus, when the nutrient supply is favorable again, the metabolic functions and cell division of the bacteria are resumed until exhaustion of the nutrients, then and once again; the bacteria begin their “hibernation period.”^15^

 The aim of this study was to investigate if the concentration, exposure time and preheating of NaOCl improved its antibacterial effect and dissolution ability on *in situ* infected dentin, and like wise test if the post-treatment biofilm was capable of growing again in a nutrient-rich medium.

## Materials and Methods

 The study protocol was approved by the Institutional Human Ethical Committee (Protocol 166/2011). The irrigant solutions used in this study were 1% and 2.5% NaOCl (Farmacia Específica, Bauru, SP, Brazil). The temperatures tested were 22ºC (room temperature) and 37ºC (body temperature), and the exposure times were 5 and 20 min. To heat the NaOCl, disposable syringes containing 1 mL of solution were placed 30 min before being used in an incubator at 37°C. A new syringe was used every 5 min. The laboratory temperature was controlled using a mercury barometer. The temperature of the solutions was controlled using a pH meter with a temperature sensor (AlfaKitLtda, Florianopolis, SC, Brazil).

 Bovine dentin blocks of 5×5×3 mm were obtained from freshly extracted bovine teeth according to a methodology previously published by the authors.^16^Samples were autoclaved for 30 min at 121ºC (Sercon-Modelo HS-Mogi das Cruzes, SP, Brazil) and then treated with 2.5% NaOCl for 15 min and 17% EDTA for 3 min. An *in situ* model was modified to induce the dentin infection.^2^

 The dentin blocks with enamel or irregular surfaces were discarded. The samples were fixed in the cavities of Hawley’s orthodontic device using sticky wax (Kota Ind. e Com. Ltda.; São Paulo, SP, Brazil). Five dentin blocks were used for each experimental procedure and four confocal ‘stacks’ were analyzed from random areas of each sample, totaling 40 samples throughout the experiment; this means that 480 images were recorded (20 pre-treatment, 20 post-treatment and 20 post- reactivation). The same samples were utilized to perform these procedures because the Live/Dead dye did not interfere with the cellular viability.^16^ The opposite side to the infected dentinal surface was marked with nail varnish to facilitate the identification of the biofilm side.

 A healthy single volunteer used the Hawley’s retainer for 48 hours. After this time, the samples were transferred to test tubes containing* *5 mL BHI broth* *and* *incubated* *at 37°C for 24 hours to standardized the bacterial growth.The subject maintained recommended oral hygiene practices.

 When the dentinal infection period was finalized, the samples were rinsed with distilled water to remove the non-adherent cells. Next, the biofilm was stained with Live/Dead BacLight Bacterial Viability (Live/Dead, Bacligth, Invitrogen, Eugene, OR, USA); in accordance with Stojicic et al.^17^The pre-NaOCl treatment samples were used as a control group. The images of the pre-treated samples were obtained and recorded using a Confocal Laser Scanning Microscope (CLSM; Leica TCS-SPE, Mannheim, Germany). Four random areas of each block were scanned using a ×40 oil lens, 1.5 μm step-size, and a format of 512 × 512 pixels. The area of each image represented 275 × 275 μm. The rank of laser penetration into biofilm was 30-80 μm. The biofilm was measured from the outer biofilm layer to the dentinal surface.

**Figure 1.**
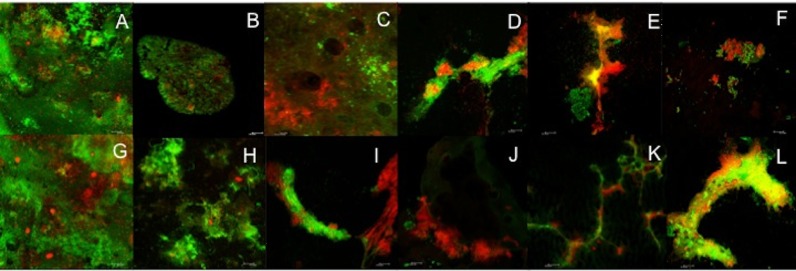
Preoperative images (A) and (G). Treated biofilm with 1%NaOCl-5min at 22ºC (B) and 37ºC (H), where a substantial amount of biomass and live cells (green) were observed. Dissolution areas (×63) (C). The dissolution and antibacterial ability improved after 20min of being exposed to 1%NaOCl at 22ºC (D) and 37ºC (I). The biofilm treated with 2.5%-NaOCl-5min at 22ºC (E) and 37ºC (J). The best antibacterial effect was found after 2.5%-NaOCl-20min (F &K), regardless of its temperature. The revitalized biofilm after treatment with 2.5%-NaOCl-20min at 37ºC (L).

 The samples were then immersed in 24-well tissue culture plates, containing 2mL of the experimental solutions for 5 min (n=5) and 20 min (n=5). The solution of 20-min groups were refreshed every 5 min to simulate the clinical conditions (the old solution was sucked and the fresh solution was added). After the contact time tests, the samples were immersed in 2 mL of 5% sodium thiosulphate for 10 min to counter the NaOCl residual effect. After that, the samples were stained again and the post-treatment biofilms were analyzed in the CLSM. The dentin blocks were then irrigated with distilled water and incubated in 5 mL of BHI at 37ºC for 24 hours. After the incubation period, the samples were stained and analyzed again to verify the bacterial reactivation. Representative images of the pre- and post-treatment samples and revitalized biofilm can be observed in Figure 1.

 The total biovolume (expressed in μm^3^/μm^2^),^18^ and the percentage of viable cells were calculated using the Bioimage_L program.^19^

 The Prisma 5.0 (GraphPad Software Inc, La Jolla, CA) was utilized as the analytical software. Statistical analysis was performed using D’Agostino-Pearson omnibus normality test to verify the normal distribution of the data. The Kruskal-Wallis and Dunn tests (P < 0.05) were used for multiples comparisons because the data did not pass the normality test.

## Results

###  Pre- and Post-treatment Biofilms

 All the experimental solutions were able to decrease the ratio of the total biovolume (P<0.05) without statistical differences among the experimental groups (P>0.05). No statistical differences were found between the percentage of live cells of the control group and 1%-NaOCl-5 min at 22ºC (P=0.09) and heated at 37ºC (P=0.08).The medians and the 25**–**75% percentiles of the total biovolume and the live cells percentage after treatment with NaOCl at 22ºC and 37ºC are shown in Table 1.

**Table 1:** Medians and 25–75% percentiles of the total biovolumeand the live cells percentage after treatment with NaOCl at room (22ºC) and body (37ºC) temperatures

**Study groups**	**Total biovolume (µm ^3 ^ /µm ^2 ^) **	**Live cells (%)**
**Control**	1.19 (0.50-3.06)^a^	94.10 (84.42-96.49)^a^
**1%–5min–22ºC**	0.05 (0.02-0.25)^b^	69.26 (37.62-87.38)^ab^
**1%–20min–22ºC**	0.04 (0.00-0.14)^b^	45.13 (13.71-84.03)^bc^
**2.5%–5min–22ºC**	0.03 (0.00-0.16)^b^	49.73 (9.63-85.07)^bc^
**2.5%–20min–22ºC**	0.01 (0.00-0.04)^b^	19.58 (3.02-62.94)^c^
**1%–5min–37ºC**	0.04 (0.01-0.20)^b^	73.82 (5.27-93.17)^ac^
**1%–20min–37ºC**	0.03 (0.00-0.19)^b^	20.13 (1.70-63.16)^c^
**2.5%–5min–37ºC**	0.05 (0.04-0.15)^b^	54.96 (46.64-74.96)^bc^
**2.5%–20min–37ºC**	0.05 (0.02-0.07)^b^	30.13 (16.31-52.09)^bc^
Different superscript letters in each column represent significant differences (n=5/group). The percentage of live cells represents the cellular viability of the remaining biofilms after treatment.		

###  Biofilm Revitalization

**Figure 2.**
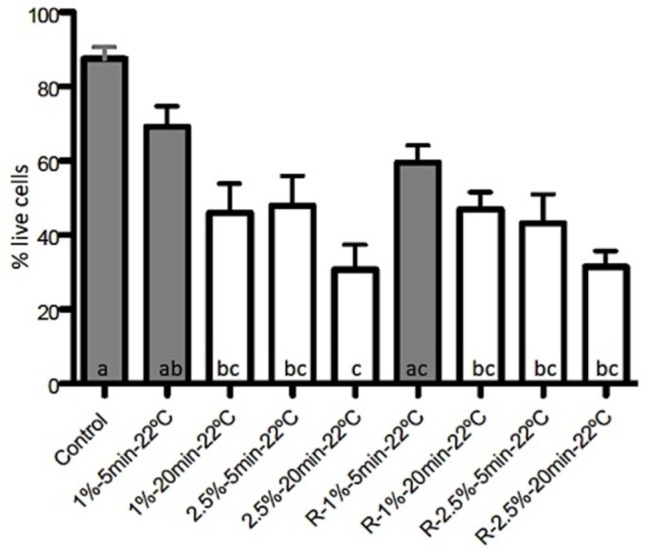
Percentage of live cells after NaOCl treatment at 22ºC. The evaluation was performed immediately or after revitalization in BHI for 24h (R). Columns represent the medians of the experimental groups. Different letters within a column depict a significant difference (P < 0.05).

 The total biovolume and percentage of live cells of the revitalized biofilm after treatment with 1% NaOCl for 5 min at room temperature showed no statistically significant differences in comparison with the control (P=0.08). Although the 1%-NaOCl at room temperature was able to significantly dissolve the organic matter (P=0.01), the remaining biofilm showed high percentages of bacterial viability (64.51%; P=0.12; Figure 2). The percentage of viable cells was not different from the control when the biofilm was exposed to 1%-NaOCl-5min at 37ºC and when this group was revitalized (63.82%) (P=0.09).

## Discussion

 There is vast information in the literature showing the effect of endodontic irrigants on single- or dual-species biofilms.^20,21^ However, the bacterial growth of a species in its natural environment profoundly differs from cells of the same species when the bacterial growth is stimulated *in vitro*.^22^ These differences are determined by variations of environmental factors such as nutrients, pH, temperature, surface and salivary flow, which are not found in biofilms developed in laboratory conditions.^23,24^

 Furthermore, mono-infections rarely occur in nature. The oral cavity is not the exception, because it presents dynamic associations of more than 500 bacterial species.^25^ Similarly, the root canal biofilm is formed by several bacterial species.^26^In line with these statements, the pathogenic power of multi-species bacterial co-aggregations is higher than the single-species biofilms.^27^ Then, testing the efficacy of the endodontic solutions on biofilms formed *in situ* would provide an appropriate correlation with *in vivo* conditions, since several bacterial species are able to survive root canal treatment or alkaline stress.^26^

 On the other hand, the dissolution and antimicrobial ability of NaOCl depends on factors such as concentration, contact time, temperature, renewal of the solution, and amount of the tissue.^2,4,5^ In the present study, all the room temperature and body temperature NaOCl groups were effective in dissolving the biofilm without statistical differences among them. These findings have similarities with a study previously published using oral biofilm formed *in situ*,^2^ in which the dissolution ability of NaOCl at room temperature was not statistically different between the 1% and 2.5% concentrations when the exposure time was 5 and 15 min. These results could be explained due to the multi-species biofilm interactions; one of them is the bacterial synergistic effect that favors the development of beneficial phenotypes such as the biofilm formation by co-aggregation, metabolic cooperation among species, and increased resistance to antimicrobial agents. These interactions are not found in mono-species biofilms.^28^

 Although, in this study, statistical differences were not found among the groups, it is worth noting that the 2.5%-NaOCl-20min at room temperature showed the highest values of biovolume reduction and antibacterial effect. These results reinforce the statement that the dissolution ability of NaOCl is directly proportional to the concentration and exposure time.^2,5^ Even so, periods of time of 15, 20, and 30 min appear to be insufficient to completely dissolve tissue and kill bacteria.^2,29^Furthermore, it was observed that the dissolution of 1%-NaOCl-37ºC for 5 and 20 min were not different than 2.5%-NaOCl-22ºC in the same exposure times, which might vaguely suggest that heating a lower concentration of NaOCl at body temperature enhanced its ability to dissolve organic matter,^5,10^ but it is very important to take these results with caution. Currently, there is no information in the literature on the dissolution effect of increasing the temperature of NaOCl using multi-species biofilms. Then, the exposure times,^2^ the lack of mechanical agitation,^5^ the presence of the exopolysaccharide matrix of the biofilms,^28^and the characteristic high variability of the bacterial volume of this type of *in situ* infection,^2,30^ could have been determining factors to explain the absence of statistical differences among the biomass of NaOCl groups, since they are able to interfere with the free chlorine dissolution ability released during heating of the hypochlorous acid present in the NaOCl.^31,32^Additionally, it has been demonstrated that the type of root canal disinfection technique, such as needle irrigation, diode laser, Photon-induced Photoacustic Streaming, sonic/ultrasonicagitation, and mechanical preparation play an important role during the root canal cleaning and shaping.^33-35^Consequently, these factors could interfere with our results and they represent the main limitations of the present study. Then, it should be considered for future investigations.

 Although the literature provides some insight into chemical stability and antimicrobial ability of NaOCl preparations, the findings appear to be somewhat contradictory. Previous publications analyzed the antimicrobial effect of the NaOCl temperature variation on uni-species biofilm^4,12^showing that the increase of the NaOCl temperature enhanced its bactericidal ability. However, the findings of Gulsahi et al^11^ showed that the irrigation time of NaOCl was more effective than the temperature (25ºC vs 37ºC) to eliminate *Enterococcus faecalis*. Similar results were observed in the present study, where the antimicrobial ability of NaOCl was more dependent on its concentration and contact time than its temperature. It is important to note that the 1%-NaOCl-5 min groups, regardless of their temperature, showed a similar percentage of live cells compared to the control group. The synergistic effects that promote bacterial biovolume, the resistance of bacteria to antimicrobial agents, and their invasion in multispecies biofilms,^36^ could be the main causes of the differences among the present study and other published investigations. Anyhow, more studies on the effect of NaOCl on polimicrobial biofilms formed *in situ* are necessary.

 Regarding the bacterial revitalization, statistical differences were not found in the present study among the NaOCl room temperature groups and their respective reactivations. The same situation was repeated in the NaOCl-37ºC groups. This would seem contradictory given that in all groups varying percentages of viable cells were found, but the slow growth rate of the bacteria within a biofilm is not necessarily due to the nutrient limitation.In fact, this slowed-down bacterial reproduction could be a response to stress conditions. This response to stress causes physiological changes that protect the bacteria from the effect of temperature and pH changes and many chemical agents.^37^ In line with this statement, Chavez de Paz et al^13^ showed that *Streptococcus anginosus* biofilm cells were able to recover 78% of the dehydrogenase activity and 61% of the esterase activity and its biomass mm^−2^ increased around 35% after 72 hours of reactivation. In addition, Shen et al^38^ using the Live/Dead technique, found that the revitalized biofilm took 4 weeks to reach a 75% bacterial growth, similar to the 3 weeks biofilms with nutrient supplementation. Considering these findings, it is possible that 24 hours is an insufficient time for the biofilm to change its stress metabolism to a rich nutrient metabolism.Consequently, the bacteria in the biofilm remained in a stationary phase.

 Based on the aforementioned, the authors concluded that an increase of the NaOCl temperature did not enhance its dissolution or antibacterial ability when it was tested on mixed-species biofilms.

## Conclusion

 Heating the NaOCl at 37ºC did not improve its dissolution ability; its antimicrobial effect is more dependent on the concentration and contact time than the temperature. The biofilm treated with NaOCl is not capable of achieving significant levels of biomass and cell viability after 24 hours of exposure to nutrient-rich conditions, except when the solution is used in low concentrations and contact times.

## Acknowledgments

 São Paulo Research Foundation (FAPESP 2011/08184-8). The authors declare that they have no competing interests.

## References

[1] Siqueira Jr JF, Paiva SS, Rocas IN (2007). Reduction in the cultivable bacterial populations in infected root canals
by a chlorhexidine-based antimicrobial protocol. J Endod.

[2] Del Carpio-Perochena AE, Bramante CM, Duarte MA, CavenagoBC CavenagoBC, Villas-Boas MH, Graeff MS (2011). Biofilm dissolution and cleaning ability of different irrigant solutions on
intraorally infected dentin. J Endod.

[3] De Sermeno RF, da Silva LA, Herrera H, Herrera H, Silva RA, Leonardo MR (2009). Tissue damage after sodium hypochlorite extrusion during root canal
treatment. Oral Surg Oral Med Oral Pathol Oral Radiol Endod.

[4] Sirtes G, Waltimo T, Schaetzle M, Zehnder M (2005). The effects of temperature on sodium hypochlorite short-term stability,
pulp dissolution capacity, and antimicrobial efficacy. J Endod.

[5] Stojicic S, Zivkovic S, Qian W, Zhang H, Haapasalo M (2010). Tissue dissolution by sodium hypochlorite: effect ofconcentration,
temperature, agitation, and surfactant. J Endod.

[6] Rossi-Fedele G, De Figueiredo JA (2008). Use of a bottle warmer to increase 4% sodium hypochlorite tissue
dissolutionability on bovine pulp. Aust Endod J.

[7] Abou-Rass M, Oglesby SW (1981). The effect soft emperature, concentration, and tissue type on the
solventability of sodium hypochlorite. J Endod.

[8] Dumitriu D, Dobre T (2015). Effects of temperature and hypochlorite concentration on the rate of
collagen dissolution. J Endod.

[9] Haapasalo M, Wang Z, Shen Y, Curtis A, Patel P, Khakpour M (2014). Tissue dissolution by a novel multisonicultra cleaning system and sodium
hypochlorite. J Endod.

[10] Cunningham WT, Balekjian AY (1980). Effect of temperature on collagen-dissolving ability of sodium hypochlorite
endodontic irrigant. Oral Surg Oral Med Oral Pathol.

[11] Gulsahi K, Tirali RE, Cehreli SB, Karahan ZC, Uzunoglu E, Sabuncuoglu B (2014). The effect of temperature and contact time of sodium hypochlorite on human
roots infected with Enterococcus faecalis and Candida albicans. Odontology.

[12] Cunningham WT, Joseph SW (1980). Effect of temperature on the bactericidal action of sodium hypochlorite
endodontic irrigant. Oral Surg Oral Med Oral Pathol.

[13] Chavez de Paz LE, Hamilton IR, Svensater G (2008). Oral bacteria in biofilms exhibit slow reactivation from nutrient
deprivation. Microbiol.

[14] Kim WS, Park JH, Ren J, Su P, Dunn NW (2001). Survival response and rearrangement of plasmid DNA of Lactococcus lactis
during long-term starvation. Appl Environ Microbiol.

[15] Navarro Llorens JM, Tormo A, Martinez-Garcia E (2010). Stationary phase in gram-negative bacteria. FEMS Microbiol Rev.

[16] Del Carpio-Perochena A, Bramante CM, de Andrade FB, Maliza AG, Cavenago BC, Marciano MA (2015). Antibacterial and dissolution ability of sodium hypochlorite in different
pHs on multi-species biofilms. Clin Oral Investig.

[17] Stojicic S, Shen Y, Qian W, Johnson B, Haapasalo M (2012). Antibacterial and smear layer removal ability of a novel irrigant,
QMiX. Int Endod J.

[18] Heydorn A, Nielsen AT, Hentzer M, Sternberg C, Givskov M, Ersboll BK (2000). Quantification of biofilm structures by the novel computer program
COMSTAT. Microbiol.

[19] Chavez de Paz LE (2009). Image analysis software based on color segmentation for characterization of
viability and physiological activity of biofilms. App Environ Microbiol.

[20] Bryce G, O'donnell D, Ready D, Ng YL, Pratten J, Gulabivala K (2009). Contemporary root canal irrigants are able to disrupt and eradicate single-
and dual-species biofilms. J Endod.

[21] Gomes BP, Ferraz CC, Vianna ME, Berber VB, Teixeira FB, Souza-Filho FJ (2001). In vitro antimicrobial activity of several concentrations of sodium
hypochlorite and chlorhexidine gluconate in the elimination of Enterococcus
faecalis. Int Endod J.

[22] Costerton JW, Cheng KJ, Geesey GG, Ladd TI, Nickel JC, Dasgupta M (1987). Bacterial biofilms in nature and disease. Annu Rev Microbiol.

[23] Coenye T, Nelis HJ (2010). In vitro and in vivo model systems tostudy microbial
biofilmformation. J Microbiol Methods.

[24] Marsh PD, Bradshaw DJ (1997). Physiological approaches to the control of oral biofilms. Adv Dent Res.

[25] Kazor CE, Mitchell PM, Lee AM, Stokes LN, Loesche WJ, Dewhirst FE (2003). Diversity of bacterial populations on the tongue dorsa of patients with
halitosis and healthy patients. J Clin Microbiol.

[26] Chavez de Paz LE (2007). Redefining the persistent infection in root canals: possible role of
biofilm communities. J Endod.

[27] Ochiai K, Kurita-Ochiai T, Kamino Y, Ikeda T (1993). Effect of co-aggregation on the pathogenicity of oral
bacteria. J Med Microbiol.

[28] Elias S, Banin E (2012). Multi-species biofilms: living with friendly neighbors. FEMS Microbiol Rev.

[29] Retamozo B, Shabahang S, Johnson N, Aprecio RM, Torabinejad M (2010). Minimum contact time and concentration of sodium hypochlorite required to
eliminate Enterococcus faecalis. J Endod.

[30] Ordinola-Zapata R, Bramante CM, Brandao Garcia R, Bombarda de Andrade F, Bernardineli N, Gomes de Moraes I (2013). The antimicrobial effect of new and conventional endodontic irrigants on
intra-orally infected dentin. Acta Odontol Scand.

[31] Frais S, Ng YL, Gulabivala K (2001). Some factors affecting the concentration of available chlorine in
commercial sources of sodium hypochlorite. Int Endod J.

[32] Van der Sluis LW, Gambarini G, Wu MK, Wesselink PR (2006). The influence of volume, type of irrigant and flushing method on removing
artificially placed dentine debris from the apical root canal during passive ultrasonic
irrigation. Int Endod J.

[33] Jonathan R, Mathew J, Suganthan P, Samuel A, John B (2013). Comparative evaluation of the antibacterial efficacy of four different
desinfection techniques in minimally instrumented experimentally infected root canals:
an in vitro study. Int J Laser Dent.

[34] Gutarts R, Nusstein J, Reader A, Beck M (2005). In vivo debridement efficacy of ultrasonic irrigation following hand-rotary
instrumentation in human mandibular molars. J Endod.

[35] Rocas IN, Lima KC, Siqueira JF, Jr Jr (2013). Reduction in bacterial counts in infected root canals after rotary or hand
nickel-titanium instrumentation--a clinical study. Int Endod J.

[36] Burmolle M, Webb JS, Rao D, Hansen LH, Sorensen SJ, Kjelleberg S (2006). Enhanced biofilm formation and increased resistance to antimicrobial agents
and bacterial invasion are caused by synergistic interactions in multispecies
biofilms. Appl Environ Microbiol.

[37] Mah TF, O'toole GA (2001). Mechanisms of biofilm resistance to antimicrobial agents. Trends Microbiol.

[38] Shen Y, Stojicic S, Haapasalo M (2010). Bacterial viability in starved and revitalized biofilms: comparison of
viability staining and direct culture. J Endod.

